# Global Research Trends in Forced Oscillation Technique (FOT) and Impulse Oscillometry System (IOS): A Bibliometric Analysis of Impact and Emerging Areas

**DOI:** 10.3390/healthcare14152336

**Published:** 2026-08-01

**Authors:** Lama O. Badghish, Afnan S. AlRaimi

**Affiliations:** Respiratory Care Department, College of Applied Medical Sciences, Imam Abdulrahman Bin Faisal University, Dammam 34221, Saudi Arabia

**Keywords:** bibliometric analysis, impulse oscillometry system, forced oscillation technique, respiratory oscillometry, trends, respiratory mechanics

## Abstract

**Highlights:**

**What are the main findings?**

- A total of 1072 publications on the Forced Oscillation Technique (FOT) and the Impulse Oscillometry System (IOS) were analyzed, demonstrating a marked global increase in oscillometry research, particularly after 2005.
- The United States of America and Australia emerged as the leading contributors, while research themes evolved from methodological foundations toward clinical applications in asthma, COPD, pediatric care, and small airway dysfunction.

**What are the implications of the main findings?**

- Bibliometric mapping identified emerging hotspots, including advanced respiratory assessment, disease monitoring, and expanding integration of oscillometry into multidisciplinary respiratory care.
- The growing number of scientific publications and international collaborations highlights the increasing clinical and research importance of oscillometry in modern respiratory medicine as a complementary assessment tool alongside traditional Pulmonary Function Tests (PFTs).

**Abstract:**

**Background/Objectives**: Assessing respiratory mechanics is essential for detecting early airway abnormalities. The Forced Oscillation Technique (FOT) and Impulse Oscillometry (IOS) have gained increasing recognition as complementary, effort-independent tools used in both research and clinical practice. This study aimed to provide a novel, comprehensive bibliometric overview of global research trends, impact, and emerging themes in FOT/IOS research. **Methods**: A systematic search of English-language articles and review articles related to FOT/IOS was conducted in the Web of Science Core Collection to identify publications related to FOT and IOS. Data were analyzed using VOSviewer, Microsoft Excel, and Biblioshiny to examine publication trends, citation patterns, collaboration networks, keyword co-occurrence, and thematic evolution. In addition, manual data extraction was performed for the top 100 most-cited papers to describe their methodological, population, and technical characteristics. **Results**: A total of 1072 studies published between 1976 and 2025 were included after screening. Research activity increased markedly after 2005, peaking in 2022 and reflecting sustained growth over time. The United States and Australia were the leading contributing countries. Frequently occurring keywords, including “Asthma”, “Forced oscillation technique”, “Impulse oscillometry”, “Lung-function”, and “Spirometry”, emerged as major research themes. Thematic development over time revealed a transition from methodological and pediatric-focused themes to more clinically relevant and practical applications, highlighting the growing clinical significance of oscillometry. **Conclusions**: Global research on oscillometry has increased significantly over the past five decades, reflecting the growing recognition of FOT and IOS as complementary tools to conventional pulmonary function tests for small airway assessment. Future research directions include continued standardization, population-specific reference values, and the evaluation of oscillometry across diverse clinical settings.

## 1. Introduction

Scientific developments in respiratory medicine have been greatly influenced by the need for measurement tools that can describe the complex mechanical dynamics of the airways with exceptional accuracy [1,2,3]. This need has driven researchers to explore techniques that detect subclinical physiological changes, characterize airway mechanics beyond the limitations of conventional lung function tests, and provide a deeper understanding of early asymptomatic airway dysfunction [1,4,5,6]. Within this scientific and technological progression, oscillometry-based methods have emerged as valuable techniques, offering sensitive and effort-independent techniques for assessing respiratory mechanics during quiet tidal breathing [6,7,8].

The forced oscillation technique (FOT) and impulse oscillometry (IOS) have gained particular prominence, progressing from fundamental physiological tools into widely used techniques for evaluating respiratory mechanics with high precision [5,7,9]. Over the past two decades, research on FOT and IOS has increased significantly due to methodological development, clinical utility, and growing recognition of their value in detecting early airway dysfunction [5,7,9,10].

Beyond its physiological applications, oscillometry has gained increasing clinical relevance due to its minimal requirements for patient cooperation, non-invasive nature, and ability to support the diagnosis and monitoring of respiratory diseases across various healthcare settings [5,7].

Despite this growing interest, the broader oscillometry research landscape, including publication patterns, key contributors, collaboration networks, and thematic evolution, remains underexplored. Furthermore, there is a lack of comprehensive evidence describing how research activity has evolved over time, which countries, institutions, and journals have driven scientific progress, how major research themes have emerged and shifted, and which highly cited studies have had the greatest influence on shaping the field, including oscillometry reference values and prediction equations.

Therefore, the purpose of this study was to perform a comprehensive bibliometric analysis to map the global development of the FOT and IOS, identify global trends, collaboration networks, and citation dynamics. Specifically, the study aimed to examine publication trends, country and institutional networks, keyword clusters, citation structures, and thematic evolution, and to highlight influential studies, key contributions, and research gaps that may guide future developments in clinical practice and respiratory research.

## 2. Materials and Methods

### 2.1. Data Collection

On 17 June 2025, we systematically searched the Web of Science Core Collection, a database indexing over 20,000 journals with rigorous indexing standards [11,12]. The Web of Science Core Collection was selected for its standardized citation indexing and widespread use in bibliometric research.

The core search strategy included the keywords: [“Impulse Oscillometry” OR “Impulse Oscillometric Technique” OR “Impulse Oscillometry System” OR “Impulse Oscillometry Testing” OR “Impulse Oscillometry-Based Assessment” OR “Oscillometry” OR “IOS” OR “Forced Oscillations” OR “FOT” OR “forced oscillation” OR “forced oscillatory impedance” OR “Forced Oscillation Technique FOT” OR “Forced Oscillatory Impedance” OR “multi-frequency FOT” OR “Frequency” OR “resonant frequency” OR “Frequency response”) AND (“Pulmonary function” OR “Lung function” OR “regional lung function” OR “Respiratory diseases”) AND (“Impedance oscillometry” OR “respiratory impedance parameters” OR “Input respiratory impedance” OR “resistance” OR “impedance” OR “reactance” OR “R5” OR “R20” OR “X5”]. These terms represented the primary focus of our investigation. Boolean operators were used to include additional synonyms, related terms, and concepts. The search was conducted using the Topic (TS) field and was restricted to English-language publications and to original research and review articles, with no restrictions on publication year.

**Figure 1:**
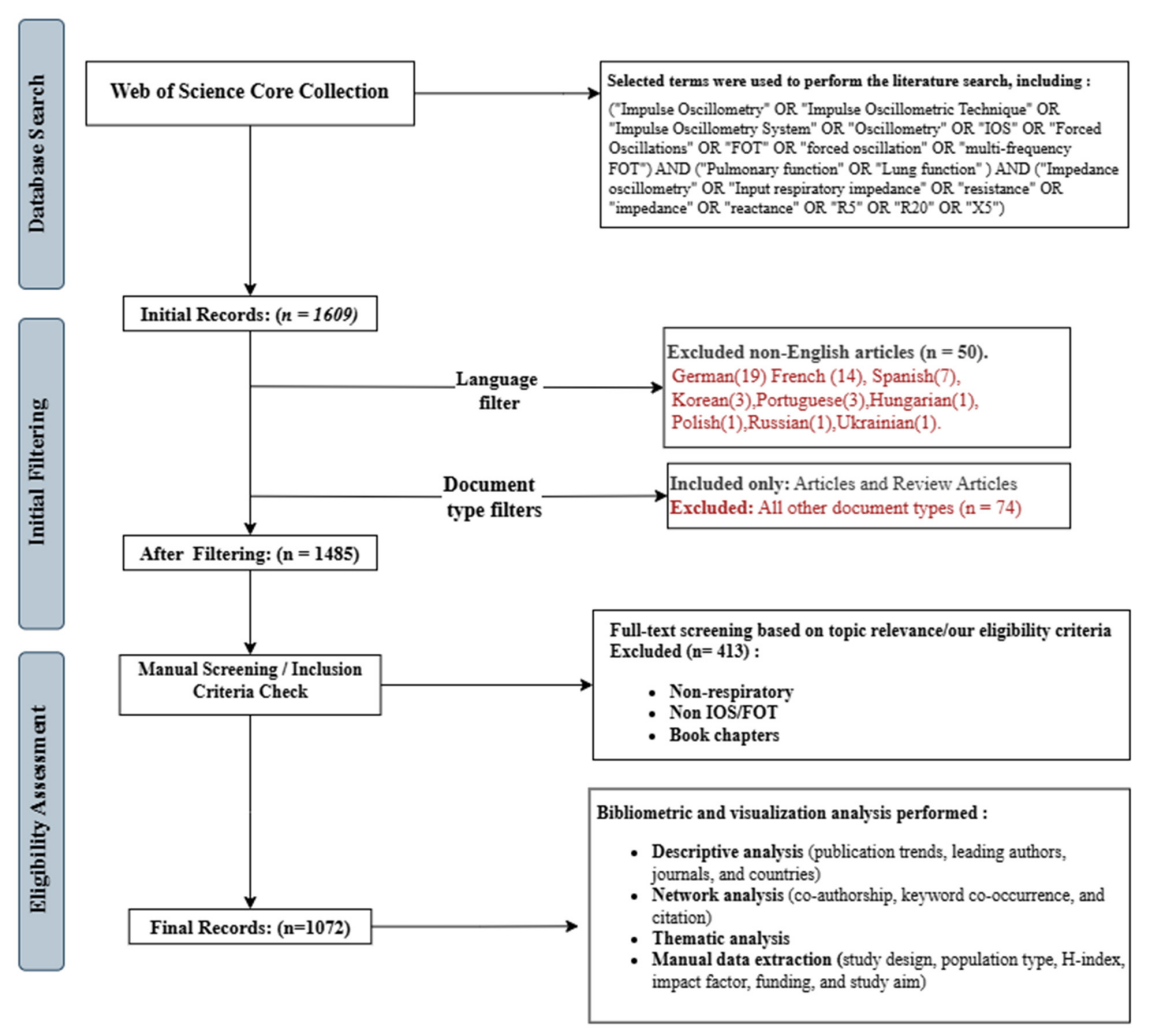
Flowchart of the study and screening process.

Initially, 1609 records were identified. After applying the predefined filters, 1485 records remained. Manual screening was then conducted, resulting in the inclusion of 1072 studies in the final analysis. The final dataset was downloaded again on 19 July 2025, in various formats (BibTeX, Excel, and plain text) to meet the requirements of different bibliometric tools and ensure reproducibility (Figure 1).

### 2.2. Data Selection Screening

To ensure the relevance and quality of the included studies, after the automated filters reduced the dataset from 1609 to 1485 records, a manual screening of titles, abstracts, and keywords for all retrieved publications was performed to assess eligibility and relevance to the study objectives. Full-text articles were reviewed when eligibility could not be determined from the title, abstract, and keywords. The manual screening process was conducted by L.O.B. using predefined eligibility criteria under the supervision of A.S.A. Records with uncertain eligibility were reviewed and discussed with the supervisor to ensure consistent inclusion decisions and the relevance of the final dataset. Studies were included if they investigated, applied, or reported FOT/IOS measurements in a clinically or scientifically relevant context. This rigorous approach ensured that only studies related to the forced oscillation technique and the impulse oscillometry system were included, maintaining the validity and focus of the dataset and analysis. Records were excluded if they were non-respiratory publications, studies unrelated to FOT/IOS measurements or applications, or book chapters, as summarized in Figure 1. This process resulted in a final dataset of 1072 publications, highlighting the growing scientific interest in oscillometry research.

### 2.3. Data Analysis

For the bibliometric analysis, VOSviewer (version 1.6.20) and Biblioshiny (bibliometrix R package, version 4.4.2) were used to perform citation analysis and generate network visualizations, including keyword co-occurrence and co-authorship maps [13,14,15]. Country productivity was assessed using the Countries’ Scientific Production analysis in Biblioshiny. Unless otherwise specified, the default analytical settings of VOSviewer and Biblioshiny were applied. Minor adjustments to the network visualization layout were made only to improve the clarity and readability of the figures.

Microsoft Excel was used for data management, duplicate checking, and manual data extraction. Extracted variables included study design, access status, population characteristics, H-index, journal impact factor, oscillometry type, funding status, and study objectives. The top 100 cited papers were selected from the final dataset according to the “Times Cited, WoS Core” metric. Manual extraction of these articles was performed to accurately classify study-specific contextual variables that automated bibliometric tools do not reliably capture and to validate the automated data. The extracted data were reviewed and cross-checked multiple times to ensure accuracy and consistency.

Furthermore, during the keyword co-occurrence analysis, minor formatting differences were recognized and standardized. Identical terms were merged to avoid duplication and improve analytical accuracy. The annual growth rate was obtained using Biblioshiny, and the coefficient of determination (R^2^) was calculated from the annual publication trend using Microsoft Excel.

## 3. Results

### 3.1. Publication Trend

**Figure 2:**
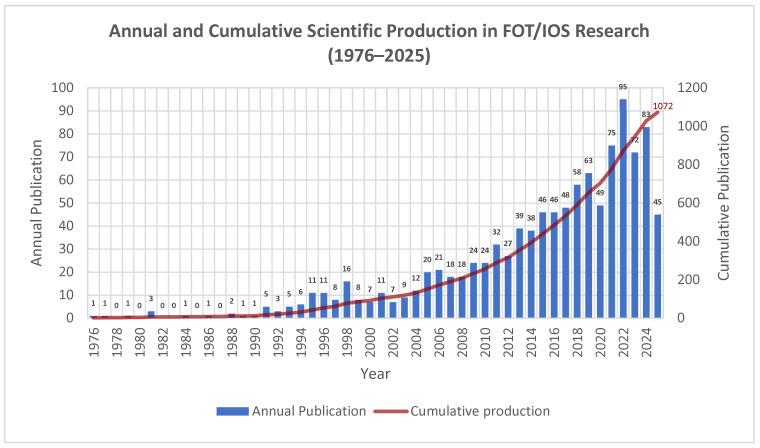
Annual and Cumulative Scientific Production in FOT/IOS Research (1976–2025).

Publication trends over time offer a useful lens for understanding the evolving research focus within the field (Figure 2). From 1976 to 2025, a total of 1072 publications on FOT/IOS were identified. The annual number of publications remained very low until the early 1990s, with fewer than five publications per year. A gradual increase was observed from the late 1990s to 2005, accompanied by significant fluctuations.

From 2005 onward, the trend accelerated markedly, with the most rapid expansion occurring between 2010 and 2022, peaking at 95 publications in 2022. Overall, the field demonstrated a sustained upward trajectory over the past five decades, supported by an annual growth rate of 8.08% and a strong correlation between publication output and time (R^2^ = 0.907).

**Figure 3:**
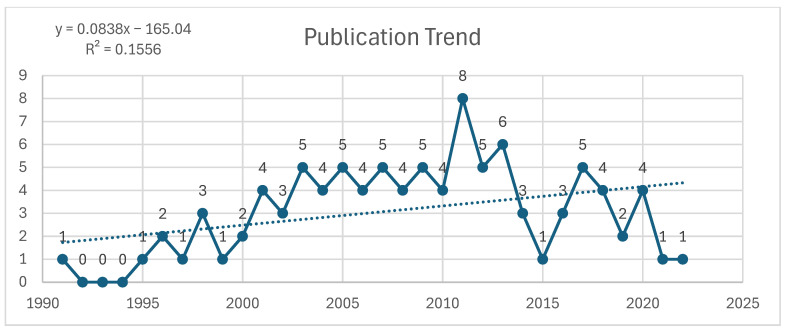
Publication trend of the 100 most-cited papers in the FOT/IOS field. The dashed line represents the linear trend of publications over time.

The publication trend of the top 100 most-cited papers is shown in Figure 3. Publications were distributed across the years from the early 1990s to 2022, with a peak in 2010 (n = 8). This wide temporal distribution helps minimize potential age-related citation bias.

### 3.2. Cumulative Productions

The cumulative growth of publications on FOT/IOS demonstrates a remarkable increase over time (Figure 2). The curve shows a steady but slow accumulation of studies until the mid-1990s, followed by a noticeable increase in the growth rate.

Subsequently, there was a remarkable increase from 2005 onward, with a notably sharp increase between 2012 and 2025. By 2025, the total number of publications had reached 1072, even though the year was still ongoing. The cumulative growth curve also demonstrated a strong fit over time (R^2^ = 0.975), indicating a steadily growing publication trend in the field.

### 3.3. Countries

**Figure 4:**
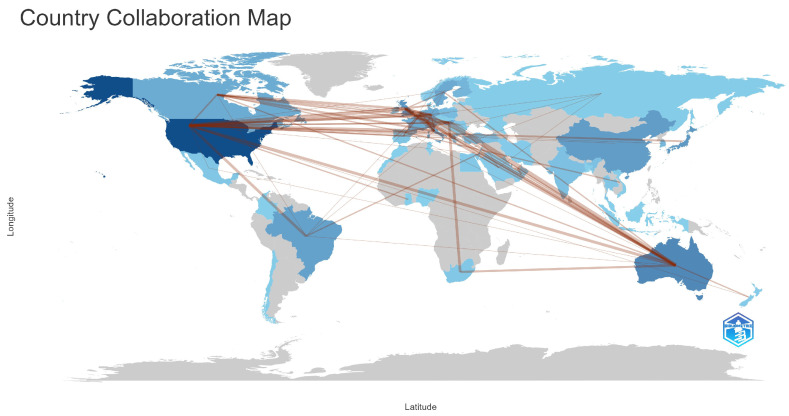
Countries’ contributions and collaboration in the field of FOT/IOS. The different shades of blue indicates varying level of research activity across countries, while connecting lines represent international collaborations.

The research output in the FOT/IOS field has been distributed across approximately 50 countries (Figure 4). The United States of America (USA) had the highest country contribution frequency (745), followed by Australia (511) and China (423) (Table 1). Because a single publication may involve authors from multiple countries, country frequencies are not expected to equal the number of included publications. Other active contributors included Brazil, Japan, Sweden, the United Kingdom, Canada, France, and Italy.

International collaboration was common. Notable collaborations included Australia-Hungary (23) and the United States-United Kingdom (19). The USA and Australia emerged as major centers linking international research networks in this field.

Notably, the Kingdom of Saudi Arabia (KSA) was identified as anitspublication output remained lower than that of the leading nations. The contribution from KSA reflects the increasing recognition of oscillometry in the region.

### 3.4. Top Cited Papers

The top 10 cited papers in the field of FOT/IOS are presented in Table 2, highlighting their significant impact. The most cited study was published by Oostveen et al. in 2003, with 962 citations, followed by Vestbo et al. in 2008, with 562 citations, and Bickel et al., with 266 citations [7,9,16]. Most of these papers were published in leading high-impact respiratory medicine journals.

After adjusting for publication year using normalized total citations (Normalized TC), the most impactful papers differed from those ranked by raw citation counts. The highest normalized TC values were recorded for Bickel et al. (9.58), Vestbo et al. (8.74), and Brashier et al. (7.99) [9,16,17] (Table 2).

**Table 2:** Top 10 cited papers in the field of FOT/IOS.

Rank	Title	1st Author (Year)	Total Citations	TC per Year	Normalized TC
1	The Forced Oscillation Technique in Clinical Practice: Methodology, Recommendations and Future Developments [7].	Oostveen E (2003)	962	41.83	5.75
2	Evaluation Of COPD Longitudinally to Identify Predictive Surrogate End-points (ECLIPSE) [16].	Vestbo J (2008)	562	31.22	8.74
3	Impulse Oscillometry [9].	Bickel S (2014)	266	22.17	9.58
4	Noninvasive And Invasive Pulmonary Function in Mouse Models of Obstructive and Restrictive Respiratory Diseases [18].	Vanoirbeek JAJ (2010)	263	16.44	7.18
5	Respiratory Impedance in Healthy Subjects: Baseline Values and Bronchodilator Response [19].	Oostveen E (2013)	196	15.08	5.92
6	Lung Function Measurement in Awake Young Children [20].	Bisgaard H (1995)	194	6.26	3.44
7	Relating Small Airways to Asthma Control by Using Impulse Oscillometry in Children [21].	Shi Y (2012)	181	12.93	5.29
8	Exhaled Nitric Oxide Rather Than Lung Function Distinguishes Preschool Children with Probable Asthma [22].	Malmberg LP (2003)	178	7.74	1.06
9	Measuring Lung Function Using Sound Waves: Role of the Forced Oscillation Technique and Impulse Oscillometry System [17].	Brashier B (2015)	178	16.18	7.99
10	Impulse Oscillometry Provides an Effective Measure of Lung Dysfunction In 4-Year-Old Children at Risk for Persistent Asthma [23].	Marotta A (2003)	172	7.48	1.03

### 3.5. Journals and Co-Cited Journals

**Figure 5:**
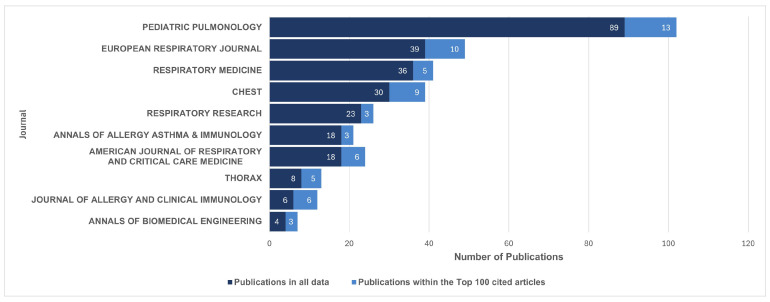
Distribution of publications by journal (all data vs. top 100 cited articles).

Two hundred eighty-nine journals were identified through bibliometric analysis as having published studies in the FOT/IOS field. The distribution of publications across journals in both the overall dataset and the top 100 most-cited articles is illustrated in Figure 5. Pediatric Pulmonology led with 89 publications and contributed 13 articles to the top 100 cited papers. It was followed by the European Respiratory Journal, with 39 total publications and 10 top-cited articles; Respiratory Medicine, with 36 total publications and 5 top-cited articles;; Respiratory Medicine, with 36 total publications and 5 top-cited articles; and CHEST, with 30 total publications and 9 top-cited articles. Other high-impact journals, such as the Journal of Allergy and Clinical Immunology and The American Journal of Respiratory and Critical Care Medicine, also contributed influential publications to the field.

### 3.6. Keyword Co-Occurrence Network

**Figure 6:**
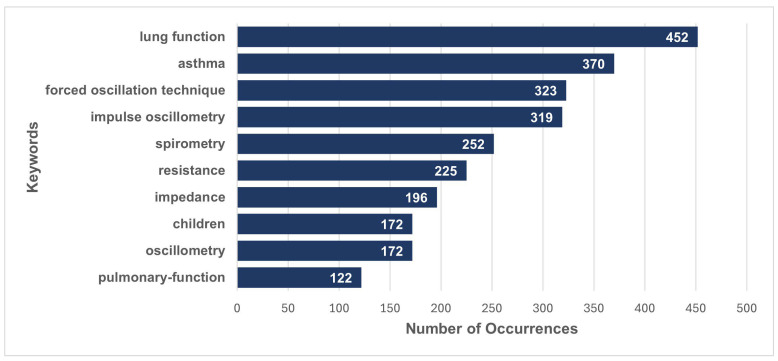
Top keywords and their occurrence frequency in the FOT/IOS field.

The keyword analysis showed several keywords that frequently occurred in FOT/IOS research (Figure 6). The top ten most frequent keywords included lung function (n = 452), asthma (n = 370), forced oscillation technique (n = 323), impulse oscillometry (n = 319), spirometry (n = 252), resistance (n = 225), impedance (n = 196), children (n = 172), oscillometry (n = 172), and pulmonary-function (n = 122).

**Figure 7:**
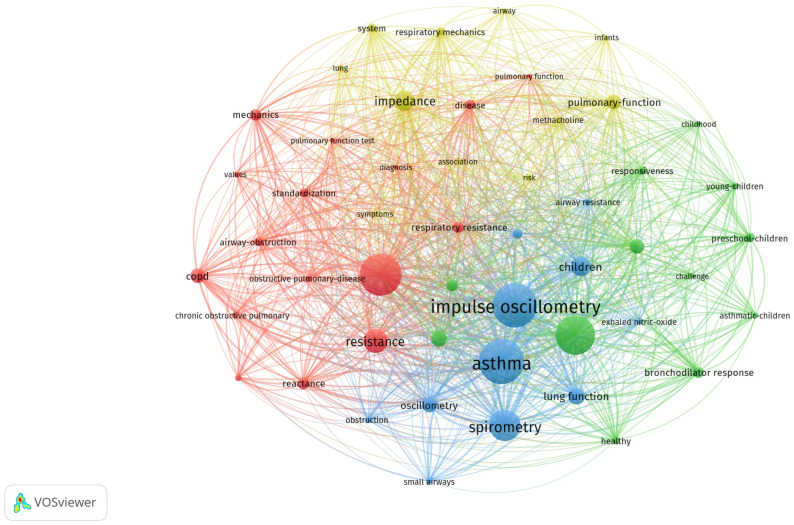
Overlay visualization of keywords in the field of FOT/IOS.

Figure 7 reveals three major thematic clusters. Cluster 1 (red) includes 12 items mainly related to respiratory mechanics and obstructive diseases, such as the forced oscillations technique, impedance, and COPD, reflecting the physiological and methodological aspects of oscillometry in airway disease assessment.

Cluster 2 (blue) includes 11 items edsuch asfocused on assessing pulmonary function and mechanics in respiratory diseases, such as asthma, spirometry, impulse oscillometry, adults, children, management, and standardization.

Cluster 3 (green) includes 11 items, such as reference value, methacholine, and bronchodilator response, highlighting research focused on assessing pulmonary function testing and airway responsiveness.

### 3.7. Thematic Evolution

**Figure 8:**
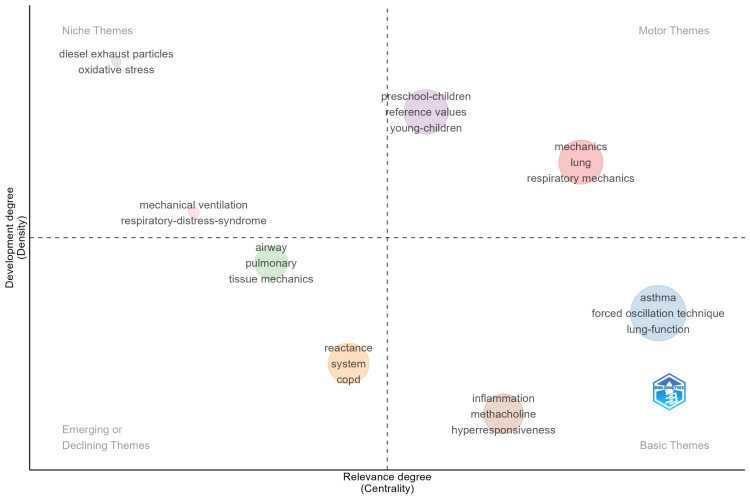
Thematic clustering of research topics from 1976 to 2018 based on centrality and density.

**Figure 9:**
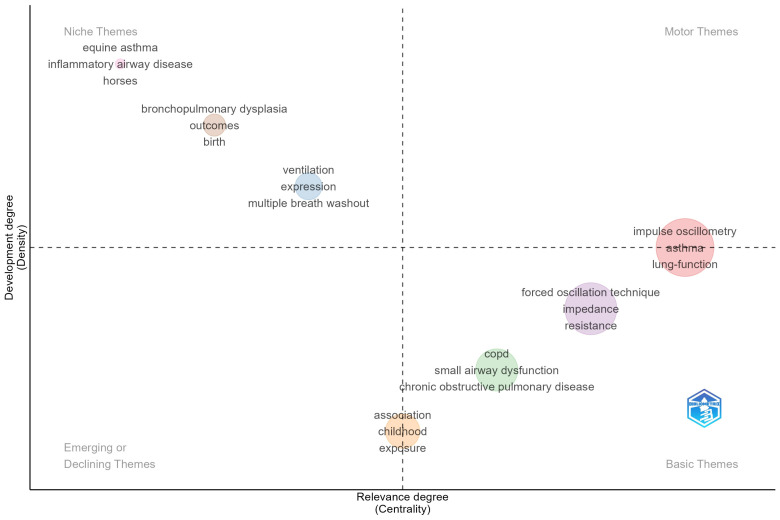
Thematic clustering of research topics from 2019 to 2025 based on centrality and density.

The thematic analysis of FOT/IOS research is represented in Figure 8 and Figure 9, covering two periods (1976–2018 and 2019–2025). During the first period (1976–2018) (Figure 8), themes such as forced oscillation technique, lung function, and asthma were represented as basic themes, forming the foundation of the field with high centrality but relatively low development. A cluster containing respiratory mechanics, lung, preschool-children, and reference values was identified as a motor theme, representing their strong internal development and an important role in methodological studies. Niche themes were represented by oxidative stress and diesel exhaust particles, whereas methacholine, respiratory distress, inflammation, hyperresponsiveness, and mechanical ventilation appeared in the declining or emerging quadrant.

In contrast, during the 2019–2025 period (Figure 9), a clear thematic shift toward clinical applications and airway assessment was observed. Impulse oscillometry, asthma, COPD, and lung function emerged as motor and basic themes, highlighting their dominance and relevance in recent studies. Emerging themes such as bronchopulmonary dysplasia, multiple breath washout, ventilation, and exposure began to appear in the declining or emerging quadrant.

### 3.8. Organizations

**Figure 10:**
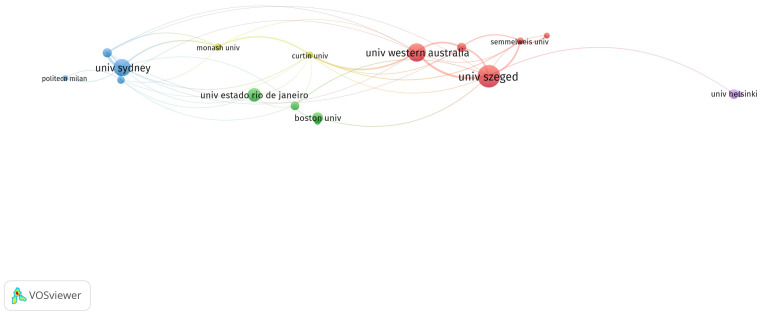
Network visualization of institutions and their collaboration. Node size reflects the relative contribution of each institution, link thickness indicates the strength of collaboration the strength of collaboration, and colors represent different collaboration clusters identified by VOSviewer.

The most productive institutions and their collaboration networks in the FOT/IOS research field are shown in Figure 10. The collaboration network formed four main distinct clusters, represented by different colors. The University of Szeged and the University of Western Australia formed the central hubs of collaboration. Both institutions demonstrated active collaborative links with Semmelweis University, Monash University, and Curtin University. This highlights the role of international research networks in shaping the field’s development and the strong partnership between Europe and Australia. Additional active contributors, such as the University of Sydney and the Woolcock Institute of Medical Research, also maintained visible collaborative links.

### 3.9. Descriptive Characteristics of the Top 100 Most-Cited Studies

**Figure 11:**
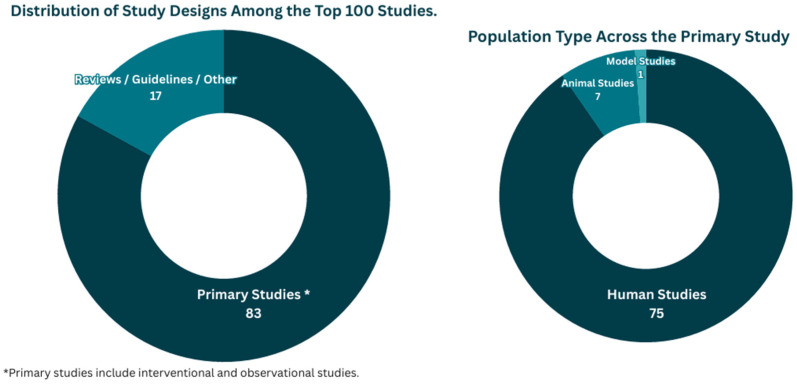
Overview of the characteristics of the 100 top-cited studies.

Manual data extraction was performed for the 100 most-cited publications to characterize their methodological, population, technical, and publication characteristics (Figure 11). The complete list of the top 100 most-cited studies is provided in the Supplementary Materials (Table S1).

Observational studies accounted for the largest proportion of the dataset, with cross-sectional and cohort designs being the most common, whereas accounted for the largest proportion of the dataset, with cross-sectional and cohort designs being the most common, whereas interventional studies were less frequent. Experimental research, primarily involving animal models (including rats, mice, and dogs) and experimental airway models under controlled conditions, accounted for a smaller proportion.

From a technical perspective, IOS was slightly more frequently used than FOT. Across the studies, airway resistance and reactance were the most reported parameters, reflecting their central role in characterizing airway mechanics.

Regarding publication characteristics, the top 100 cited papers were distributed across both open-access and closed/subscription-based journals, with no clear dominance of one type over the other. Most studies were published in journals with a wide range of impact factors.

## 4. Discussion

The global research landscape of respiratory oscillometry has shown remarkable progress over the past five decades. The field has evolved from a niche methodological concept into a recognized area within respiratory research, with growing clinical applications [6,9,24]. This evolution is reflected in the steady and significant increase in publication output, particularly after the early 2000s. This shift is not limited to numerical growth. Oscillometry is increasingly cited in pulmonary research and applied in disease-specific studies. At the same time, efforts toward clinical standardization have expanded, including the development of reference values, measurement protocols, and technical standards to support its use in practice [6,25,26].

This bibliometric analysis identified several important findings and demonstrated clear growth in oscillometry research over time. A noticeable increase in publication activity occurred after 2005, reflecting growing interest in and recognition of noninvasive, sensitive respiratory assessment methods in both research and clinical practice [27,28,29].

The cumulative growth pattern in publications on FOT/IOS reflects the technological and methodological progress within the field. The marked rise after the 2000s could be associated with improvements in device technology, advances in signal-processing methods, and the publication of key standardization documents that enhanced the clinical relevance and reliability of oscillometry [30]. Global research activity expanded as oscillometry became increasingly recognized for its ability to assess respiratory mechanics, particularly in small airway dysfunction [31,32]. This trend also reflects a broader shift toward noninvasive, effort-independent diagnostic approaches [29].

The geographical distribution of publications shows that both established and emerging contributors have shaped oscillometry research. The USA, followed by Australia and China, has been among the leading contributors to the field [6]. This pattern may reflect the presence of well-established respiratory research centers and early adoption of oscillometry in both methodological and clinical research [6,7].

The participation of emerging contributors, such as Egypt and the KSA, highlights the growing international recognition of oscillometry and its increasing relevance in the global respiratory care field [33]. Despite its limited clinical application in KSA, growing research activity suggests increasing interest that may lead to wider acceptance in the future. The availability of commercial oscillometry systems may have supported research activity in the field. Despite this accessibility, oscillometry is still not widely implemented in routine clinical practice [33].

The importance of early methodological studies in shaping the development of FOT/IOS is reflected in their strong presence among the most-cited papers. The study by Oostveen et al. (2003) provided comprehensive methodological recommendations and established the technical foundations that later studies continued to refine, and it remains a key reference for the standardization and clinical integration of oscillometry [7].

Although landmark studies dominate total citation counts, the normalized TC analysis shows that more recent publications continue to contribute to the development of oscillometry. Papers such as Bickel et al. (2014), Vestbo et al. (2008), and Brashier et al. (2015) have helped refine methodological understanding and support the clinical use of oscillometry [9,16,17]. Importantly, the distribution of top-cited papers across both early and more recent years suggests a reduction in age-related citation bias and reflects that their impact is driven primarily by the strength of their methodological contributions rather than by the age of publication [34].

The thematic structure derived from keyword analysis provides important insights into the conceptual and clinical structure of oscillometry over time. One of the major themes highlights the field’s strong physiological and methodological foundations, emphasizing the role of oscillometry in describing respiratory mechanics and airway disorders [30,35]. Additional themes indicate the broad clinical use of IOS across different age groups and respiratory conditions, often in combination with spirometry in both clinical and research settings. The presence of themes related to reference values and airway responsiveness further reflects ongoing efforts toward standardization and the integration of oscillometry into PFT protocols [6,36].

Together, these patterns indicate that oscillometry research has expanded to include methodological progression, therapeutic and clinical relevance, and efforts toward diagnostic standardization [6,37]. This progression reflects a field that continues to evolve in both conceptual and clinical aspects.

The thematic analysis shows a clear evolution of scientific priorities and a shift in the focus of oscillometry research over time. Earlier studies between 1976 and 2018 primarily focused on methodological development, respiratory mechanics, and foundational clinical concepts. These themes reflect the early phase of the field, where the primary objectives were to develop the physiological foundations, measurement principles, and technical reliability of FOT and IOS [7,8].

In contrast, research published between 2019 and 2025 shows a significant shift toward more clinically focused, age- and population-specific applications, highlighting the need to establish more specific reference values [28,35,38]. The emergence of themes such as bronchopulmonary dysplasia and multiple breath washout reflects the expanding integration of oscillometry into neonatal and pediatric respiratory assessment [37,39]. Similarly, the prominence of impulse oscillometry, COPD, resistance, and impedance as important themes indicates increasing clinical use and recognition of oscillometry as a sensitive tool for detecting small airway dysfunction and monitoring disease progression [38,40].

Collectively, these patterns indicate that the field has progressed from establishing technical foundations to the clinical implementation of oscillometry, including diagnostic, monitoring, and population-specific applications [28,41,42,43].

Technology acceptance frameworks, such as the Technology Acceptance Model (TAM) and its extensions, may offer insight into the growing interest in oscillometry [44]. These frameworks emphasize that user adoption is influenced by perceived usefulness and perceived ease of use, while extended models also consider additional factors such as social influence and compatibility [44]. In scientific and clinical contexts, these factors often evolve as the practical value of technology becomes clearer to the research community and evidence accumulates [45,46].

Early recommendations for the use of oscillometry were published in 2003, followed by a gradual increase in research activity around 2005 [7]. The evidence base for oscillometry expanded through an increasing number of original studies and review articles published between 2003 and 2018 [45]. During this period, research increasingly emphasized the limitations of conventional spirometry in specific patient populations while demonstrating the clinical utility and sensitivity of oscillometry for detecting small airway dysfunction [4,17,19]. As evidence accumulated and the value of the technique became more widely recognized, the research focus gradually shifted toward clinical applications, with this transition becoming most evident around 2018.

The ERS Technical Standards published in 2020 introduced standardized procedures, such as quality control, artifact handling, and reference values, which likely improved confidence in the technique and facilitated its broader acceptance [6]. This may account for the continued growth of oscillometry research and the peak in publications observed in 2022 [6,46]. These developments have strengthened the role of oscillometry as a reliable complementary tool for respiratory assessment [6,47].

The predominance of observational studies among the top-cited papers reflects the practical utility of oscillometry as a clinical tool [35]. FOT and IOS require minimal patient cooperation, integrate smoothly into routine clinical assessment, and are suitable for research that reflects real clinical practice [5,30]. This has allowed researchers to evaluate the performance and sensitivity of oscillometry across various patient populations and disease severities, providing insights into its practical strengths and limitations beyond controlled laboratory settings [48].

The use of animal models, including rats, mice, and dogs, reflects the important role of preclinical research in establishing a physiological basis before integration into human studies [18,49]. These experimental models provided a controlled environment for characterizing airway mechanics, validating measurements, and understanding how oscillatory signals behave within the respiratory system [18,50]. Although the number of studies using experimental models was relatively limited, they illustrate the technical efforts that contributed to refining the measurement and validation of fundamental oscillometric parameters and their clinical application [49].

The slightly higher use of IOS in the top 100 most-cited papers likely reflects its greater commercial availability, as IOS devices are more widely used in clinical settings compared with traditional FOT systems [1,49]. This broader accessibility has facilitated its integration into clinical practice, especially in pediatric and elderly populations [51]. Regardless of the oscillometry technology used, the consistent reporting of resistance and reactance across studies highlights the essential role of these parameters in describing airway mechanics [5,6,52].

The presence of highly cited studies across both open- and closed-access journals suggests that accessibility alone is not the only factor influencing citation impact in oscillometry research [34]. Methodological rigor and clinical relevance seem to be more important factors in determining scientific influence, even though open access generally increases visibility and global reach [34,53]. This balance demonstrates that both publication models continue to play important roles in shaping the distribution and recognition of high-impact research [34].

### 4.1. Relevance to Research and Practice

Current research trends in oscillometry suggest several advanced hypotheses and future research directions that require further investigation. First, a broader evaluation of recent developments in oscillometry research suggests potential opportunities for integrating emerging technologies such as machine learning and artificial intelligence into predictive models that integrate oscillometric parameters for more accurate and individualized assessments.

Second, longitudinal and interventional studies are needed to explore the prognostic value of oscillometry in disease monitoring, treatment response, and long-term outcomes.

Third, integrating oscillometry with other non-invasive assessment tools, such as fractional exhaled nitric oxide (FeNO), multiple-breath nitrogen washout (MBNW), and body plethysmography, may provide a more comprehensive understanding of small airway dysfunction and respiratory mechanics.

Finally, defining population-specific reference values for both FOT and IOS remains essential, especially considering variations among age groups, physiological characteristics, and device types. In addition, reference equations may be both device- specific and population-specific. Therefore, the interpretation of FOT/IOS measurements should consider the equipment model, software version, testing protocol, and quality control standards, as these factors may influence measured values and limit direct comparability across studies and clinical settings.

Collectively, these directions highlight the growing scientific interest in advancing oscillometry toward more precise and clinically integrated applications.

### 4.2. Study Strengths and Limitations

This study has several significant strengths. It provides a global overview of oscillometry research, including geographical, institutional, and thematic analyses that enhance the understanding of global scientific efforts in this field. Moreover, the inclusion and manual analysis of the top 100 most-cited publications improved the dataset’s accuracy and reliability, thereby enhancing the validity of the findings.

Several limitations should also be acknowledged. The analysis was restricted to the Web of Science Core Collection and English-language publications, which may have resulted in the exclusion of relevant publications indexed in other databases or published in other languages. The use of a single database may have influenced the overall publication coverage and bibliometric findings. Moreover, variations in author names, despite attempts at standardization, may have resulted in minor inconsistencies. Citation-based analysis may also favor older publications that have had more time to accumulate citations. In addition, publications from 2025 may be underrepresented because the year was not yet complete at the time of data collection. These limitations are unlikely to affect the primary overall trends and patterns identified in this study.

## 5. Conclusions

This comprehensive bibliometric analysis provides novel insights into global research activity in oscillometry, emphasizing the evolution of the field from early methodological development to clinical applications. To our knowledge, this is the first study to systematically map the global research landscape of oscillometry, offering an overview of methodological development, clinical growth, and collaboration patterns within the field. The analysis also identifies research gaps and future research directions. While the present bibliometric analysis provides valuable insights into research productivity, influential publications, and emerging trends in oscillometry research, it does not directly evaluate the diagnostic accuracy or clinical effectiveness of FOT/IOS.

Clinically, the findings highlight the growing recognition of oscillometry as a complementary diagnostic tool alongside traditional pulmonary function testing. Although spirometry remains the primary tool for routine assessment, the increasing presence of FOT and IOS in research suggests their expanding role in respiratory assessment across different patient populations.

The identified bibliometric trends highlight standardization, the development of population- specific reference values, and the continued evaluation of oscillometry across different clinical settings as important priorities for future research.

## Figures and Tables

**Table 1:** Top 10 Contributing Countries by Author-Affiliation Frequency.

Rank	Country	Frequency	Rank	Country	Frequency
1	United States	745	6	Sweden	243
2	Australia	511	7	United Kingdom	231
3	China	423	8	Canada	199
4	Brazil	336	9	France	194
5	Japan	270	10	Italy	191

## Data Availability

The bibliometric data used in this study were retrieved from the Web of Science Core Collection database. The datasets generated and analyzed during the current study are available from the corresponding author upon reasonable request.

## Supplementary Materials

The following supporting information can be downloaded at: https://www.mdpi.com/article/10.3390/healthcare14152336/s1, Table S1: 100 most-cited publications.

## Abbreviations

The following abbreviations are used in this manuscript:

FOT	Forced Oscillation Technique
IOS	Impulse Oscillometry System
COPD	Chronic Obstructive Pulmonary Disease
WoS	Web of Science
AI	Artificial Intelligence
ERS	European Respiratory Society
