# Supplementary material for: Global Research Trends in Forced Oscillation Technique (FOT) and Impulse Oscillometry System (IOS): A Bibliometric Analysis of Impact and Emerging Areas
Source: Healthcare (Basel). 2026 Aug 1;14(15):2336. doi: 10.3390/healthcare14152336 (PMC13464798; doi:10.3390/healthcare14152336)
Supplement: Supplementary file 1 [file healthcare-14-02336-s001.zip › Table S1.pdf]

Rank	Title	First Author (Year)	Times Cited, WoS Core	Source Title
1	The forced oscillation technique in clinical practice:: methodology, recommendations and future developments	Oostveen, E 2003	962	EUROPEAN RESPIRATORY JOURNAL
2	Evaluation of COPD longitudinally to identify predictive surrogate end-points (ECLIPSE)	Vestbo, J 2008	562	EUROPEAN RESPIRATORY JOURNAL
3	Impulse Oscillometry Interpretation and Practical Applications	Bickel, S 2014	266	CHEST
4	Noninvasive and Invasive Pulmonary Function in Mouse Models of Obstructive and Restrictive Respiratory Diseases	Vanoirbeek, JAJ 2010	263	AMERICAN JOURNAL OF RESPIRATORY CELL AND MOLECULAR BIOLOGY
5	Respiratory impedance in healthy subjects: baseline values and bronchodilator response	Oostveen, E 2013	196	EUROPEAN RESPIRATORY JOURNAL
6	Lung function measurement in awake young children	Bisgaard, H 1995	194	EUROPEAN RESPIRATORY JOURNAL
7	Relating small airways to asthma control by using impulse oscillometry in children	Shi, YX 2012	181	JOURNAL OF ALLERGY AND CLINICAL IMMUNOLOGY
8	Measuring lung function using sound waves: role of the forced oscillation technique and impulse oscillometry system	Brashier, B 2015	178	BREATHE
9	Exhaled nitric oxide rather than lung function distinguishes preschool children with probable asthma	Malmberg, LP 2003	178	THORAX
10	Impulse oscillometry provides an effective measure of lung dysfunction in 4-year-old children at risk for persistent asthma	Marotta, A 2003	172	JOURNAL OF ALLERGY AND CLINICAL IMMUNOLOGY
11	Biofeedback treatment for asthma	Lehrer, PM 2004	171	CHEST

Rank	Title	First Author (Year)	Times Cited, WoS Core	Source Title
12	Impulse oscillometry in the evaluation of diseases of the airways in children	Komarow, HD;2011	164	ANNALS OF ALLERGY ASTHMA & IMMUNOLOGY
13	Clinical application of forced oscillation	Goldman, MD 2001	148	PULMONARY PHARMACOLOGY & THERAPEUTICS
14	Specific airway resistance, interrupter resistance, and respiratory impedance in healthy children aged 2-7 years	Klug, B 1998	146	PEDIATRIC PULMONOLOGY
15	Oscillation Mechanics of the Respiratory System	Bates, JHT 2011	145	COMPREHENSIVE PHYSIOLOGY
16	Measurement of lung function in awake 2-4-year-old asthmatic children during methacholine challenge and acute asthma: A comparison of the impulse oscillation technique, the interrupter technique, and transcutaneous measurement of oxygen versus whole-body plethysmography	Klug, B 1996	143	PEDIATRIC PULMONOLOGY
17	High-Dose N-Acetylcysteine in Stable COPD The 1-Year, Double-Blind, Randomized, Placebo-Controlled HIACE Study	Tse, HN 2013	139	CHEST
18	Postural changes in lung volumes and respiratory resistance in subjects with obesity	Watson, RA 2005	139	JOURNAL OF APPLIED PHYSIOLOGY
19	An Official American Thoracic Society Workshop Report: Optimal Lung Function Tests for Monitoring Cystic Fibrosis, Bronchopulmonary Dysplasia, and Recurrent Wheezing in Children Less Than 6 Years of Age	Rosenfeld, M2013	138	ANNALS OF THE AMERICAN THORACIC SOCIETY
20	Respiratory health in prematurely born preschool children with and without bronchopulmonary dysplasia	Vrijlandt, EJLE 2007	135	JOURNAL OF PEDIATRICS
21	The Nonallergic Asthma of Obesity A Matter of Distal Lung Compliance	Al-Alwan, A 2014	129	AMERICAN JOURNAL OF RESPIRATORY AND CRITICAL CARE MEDICINE
22	Distal airway function in symptomatic subjects with normal spirometry following world trade center dust exposure	Oppenheimer, BW 2007	128	CHEST

Rank	Title	First Author (Year)	Times Cited, WoS Core	Source Title
23	Bronchodilator response in 3-6.5 years old healthy and stable asthmatic children	Hellinckx, J 1998	121	EUROPEAN RESPIRATORY JOURNAL
24	Six-Year Follow-up of a Trial of Antenatal Vitamin D for Asthma Reduction	Litonjua, AA 2020	120	NEW ENGLAND JOURNAL OF MEDICINE
25	Evaluation of impulse oscillation system: comparison with forced oscillation technique and body plethysmography	Hellinckx, J 2001	120	EUROPEAN RESPIRATORY JOURNAL
26	Obstructive lung disease in children with mild to severe BPD	Broström, EB 2010	119	RESPIRATORY MEDICINE
27	Lung mechanics and dyspnea during exacerbations of chronic obstructive pulmonary disease	Stevenson, NJ 2005	119	AMERICAN JOURNAL OF RESPIRATORY AND CRITICAL CARE MEDICINE
28	Clinical significance and applications of oscillometry	Kaminsky, DA 2022	116	EUROPEAN RESPIRATORY REVIEW
29	Peripheral airway impairment measured by oscillometry predicts loss of asthma control in children	Shi, YX 2013	116	JOURNAL OF ALLERGY AND CLINICAL IMMUNOLOGY
30	Determinants of respiratory system input impedance and bronchodilator response in healthy Finnish preschool children	Malmberg, LP 2002	115	CLINICAL PHYSIOLOGY AND FUNCTIONAL IMAGING
31	The preschool respiratory assessment measure (PRAM): A responsive index of acute asthma severity	Chalut, DS 2000	115	JOURNAL OF PEDIATRICS
32	Respiratory symptoms and physiologic assessment of ironworkers at the World Trade Center disaster site	Skloot, G 2004	113	CHEST

Rank	Title	First Author (Year)	Times Cited, WoS Core	Source Title
33	Effect of inhaled corticosteroids on small airways in asthma: Investigation using impulse oscillometry	Yamaguchi, M2009	112	PULMONARY PHARMACOLOGY & THERAPEUTICS
34	The case for impulse oscillometry in the management of asthma in children and adults	Galant, SP 2017	111	ANNALS OF ALLERGY ASTHMA & IMMUNOLOGY
35	Adenovirus pneumonia in infants and factors for developing bronchiolitis obliterans: A 5-year follow-up	Castro-Rodriguez, JA 2006	111	PEDIATRIC PULMONOLOGY
36	ADVERSE-EFFECTS OF THE INDOOR ENVIRONMENT ON RESPIRATORY HEALTH IN PRIMARY-SCHOOL CHILDREN	CUIJPERS, CEJ 1995	100	ENVIRONMENTAL RESEARCH
37	Invasive and noninvasive methods for studying pulmonary function in mice	Glaab, T 2007	99	RESPIRATORY RESEARCH
38	Forced expiration measurements in mouse models of obstructive and restrictive lung diseases	Devos, FC 2017	98	RESPIRATORY RESEARCH
39	A study of the use of impulse oscillometry in the evaluation of children with asthma: Analysis of lung parameters, order effect, and utility compared with spirometry	Komarow, HD 2012	98	PEDIATRIC PULMONOLOGY
40	Impulse oscillometry - Reference values in children 100 to 150 cm in height and 3 to 10 years of age	Frei, J 2005	98	CHEST
41	BRONCHOCONSTRICTION AND AIRWAY HYPERRESPONSIVENESS AFTER OVALBUMIN INHALATION IN SENSITIZED MICE	HESSEL, EM 1995	96	EUROPEAN JOURNAL OF PHARMACOLOGY-ENVIRONMENTAL TOXICOLOGY AND PHARMACOLOGY SECTION
42	Impulse oscillometry: The state-of-art for lung function testing	Desiraju, K 2016	95	LUNG INDIA
43	What Does Airway Resistance Tell Us About Lung Function?	Kaminsky, DA 2012	93	RESPIRATORY CARE
44	Decreased lung function after preschool wheezing rhinovirus illnesses in children at risk to develop asthma	Guilbert, TW 2011	93	JOURNAL OF ALLERGY AND CLINICAL IMMUNOLOGY

Rank	Title	First Author (Year)	Times Cited, WoS Core	Source Title
45	Impulse oscillometry in COPD: Identification of measurements related to airway obstruction, airway conductance and lung volumes	Kolsum, U 2009	93	RESPIRATORY MEDICINE
46	Acute Effects of Electronic Cigarette Inhalation on the Vasculature and the Conducting Airways	Antoniewicz, L 2019	92	CARDIOVASCULAR TOXICOLOGY
47	Utility of impulse oscillometry in young children with asthma	Song, TW 2008	91	PEDIATRIC ALLERGY AND IMMUNOLOGY
48	Reference values for respiratory system impedance by using impulse oscillometry in children aged 2-11 years	Dencker, M 2006	91	CLINICAL PHYSIOLOGY AND FUNCTIONAL IMAGING
49	Within- and between-day variability of respiratory impedance, using impulse oscillometry in adolescent asthmatics	Goldman, MD 2002	91	PEDIATRIC PULMONOLOGY
50	Relations Between Fractional-Order Model Parameters and Lung Pathology in Chronic Obstructive Pulmonary Disease	Ionescu, CM 2009	88	IEEE TRANSACTIONS ON BIOMEDICAL ENGINEERING
51	Impulse oscillometry: A measure for airway obstruction	Vink, GR 2003	88	PEDIATRIC PULMONOLOGY
52	Guidelines for mechanical lung function measurements in psychophysiology	Ritz, T 2002	87	PSYCHOPHYSIOLOGY
53	Impulse oscillometry versus spirometry in a long-term study of controller therapy for pediatric asthma	Larsen, GL 2009	85	JOURNAL OF ALLERGY AND CLINICAL IMMUNOLOGY
54	Respiratory input impedance measurement: forced oscillation methods	MacLeod, D 2001	84	AMERICAN JOURNAL OF VETERINARY RESEARCH
55	Serial lung function and responsiveness in cystic fibrosis during early childhood	Nielsen, KG 2004	83	MEDICAL & BIOLOGICAL ENGINEERING & COMPUTING
56	Respiratory impedance and bronchodilator responsiveness in healthy children aged 2-13 years	Calogero, C 2013	82	AMERICAN JOURNAL OF RESPIRATORY

Rank	Title	First Author (Year)	Times Cited, WoS Core	Source Title
				AND CRITICAL CARE MEDICINE
57	Obesity Is a Determinant of Asthma Control Independent of Inflammation and Lung Mechanics	Farah, CS 2011	82	PEDIATRIC PULMONOLOGY
58	Pulmonary function in sickle cell disease with or without acute chest syndrome	Santoli, F 1998	82	CHEST
59	Cardiopulmonary effects of overnight indoor air filtration in healthy non-smoking adults: A double-blind randomized crossover study	Cui, XX 2018	81	EUROPEAN RESPIRATORY JOURNAL
60	Assessment of bronchodilator responsiveness in preschool children using forced oscillations	Thamrin, C2007	81	ENVIRONMENT INTERNATIONAL
61	Correlation of forced oscillation technique in preschool children with cystic fibrosis with pulmonary inflammation	Brennan, S 2005	81	THORAX
62	COGNITIVE FUNCTION AND ASSESSMENT OF LUNG-FUNCTION IN THE ELDERLY	CARVALHAESNETO, N 1995	81	THORAX
63	Quantitative computed tomography detects peripheral airway disease in asthmatic children	Jain, N 2005	77	AMERICAN JOURNAL OF RESPIRATORY AND CRITICAL CARE MEDICINE
64	Deposition efficiency of inhaled particles (15-5000 nm) related to breathing pattern and lung function: an experimental study in healthy children and adults	Rissler, J 2017	75	PEDIATRIC PULMONOLOGY
65	Respiratory function in healthy young children using forced oscillations	Hall, GL 2007	75	PARTICLE AND FIBRE TOXICOLOGY
66	Development and characterization of a rat model of chronic obstructive pulmonary disease (COPD) induced by sidestream cigarette smoke	Zheng, HG 2009	71	THORAX
67	Emotion and pulmonary function in asthma: Reactivity in the field and relationship with laboratory induction of emotion	Ritz, T 2000	71	TOXICOLOGY LETTERS
68	Case-Control Study of Lung Function in World Trade Center Health Registry Area Residents and Workers	Friedman, SM 2011	69	PSYCHOSOMATIC MEDICINE
69	Phenotypic comparison between smoking and non-smoking chronic obstructive pulmonary disease	Salvi, SS 2020	68	AMERICAN JOURNAL OF RESPIRATORY

Rank	Title	First Author (Year)	Times Cited, WoS Core	Source Title
				AND CRITICAL CARE MEDICINE
70	Small Airway Dysfunction by Impulse Oscillometry in Symptomatic Patients with Preserved Pulmonary Function	Chiu, HY 2020	68	RESPIRATORY RESEARCH
71	Determinants of early-life lung function in African infants	Gray, D 2017	68	JOURNAL OF ALLERGY AND CLINICAL IMMUNOLOGY-IN PRACTICE
72	Lung function and bronchodilator response in 4-year-old children with different wheezing phenotypes	Oostveen, E 2010	67	THORAX
73	Technique to determine inspiratory impedance during mechanical ventilation: Implications for flow limited patients	Kaczka, DW 1999	67	EUROPEAN RESPIRATORY JOURNAL
74	Relationship between peripheral airway function and patient-reported outcomes in COPD: a cross-sectional study	Haruna, A 2010	66	ANNALS OF BIOMEDICAL ENGINEERING
75	Analysis of Regional Mechanics in Canine Lung Injury Using Forced Oscillations and 3D Image Registration	Kaczka, DW 2011	64	BMC PULMONARY MEDICINE
76	Reference Values of Impulse Oscillometric Lung Function Indices in Adults of Advanced Age	Schulz, H2013	63	ANNALS OF BIOMEDICAL ENGINEERING
77	Methacholine challenge in young children as evaluated by spirometry and impulse oscillometry	Schulze, J 2012	63	PLOS ONE
78	Colored 3-Dimensional Analyses of Respiratory Resistance and Reactance in COPD and Asthma	Mori, K 2011	62	RESPIRATORY MEDICINE
79	Exercise-induced changes in respiratory impedance in young wheezy children and nonatopic controls	Malmberg, LP 2008	62	COPD-JOURNAL OF CHRONIC OBSTRUCTIVE PULMONARY DISEASE
80	Measurement of respiratory resistance in the emergency department - Feasibility in young children with acute asthma	Ducharme, FM 1997	62	PEDIATRIC PULMONOLOGY

Rank	Title	First Author (Year)	Times Cited, WoS Core	Source Title
81	Intrauterine growth retardation and lung function of very prematurely born young people	Harris, C 2021	60	CHEST
82	Lung function after extremely preterm birthA population-based cohort study (EXPRESS)	Thunqvist, P 2018	59	PEDIATRIC PULMONOLOGY
83	The Relationship Between Airflow Obstruction, Emphysema Extent, and Small Airways Function in COPD	Timmins, SC 2012	59	PEDIATRIC PULMONOLOGY
84	Lung function tests in neonates and infants with chronic lung disease: Lung and chest-wall mechanics	Gappa, M 2006	58	CHEST
85	Tobacco smoke exposure in early life and adolescence in relation to lung function	Thacher, JD 2018	57	PEDIATRIC PULMONOLOGY
86	Clinical characteristics of adult asthma associated with small airway dysfunction	Kjellberg, S 2016	57	EUROPEAN RESPIRATORY JOURNAL
87	Expression of smooth muscle and extracellular matrix proteins in relation to airway function in asthma	Slats, AM 2008	57	RESPIRATORY MEDICINE
88	Function of pulmonary neuronal M2 muscarinic receptors in stable chronic obstructive pulmonary disease	On, LS 2001	57	JOURNAL OF ALLERGY AND CLINICAL IMMUNOLOGY
89	Influence of distinct asthma phenotypes on lung function following weight loss in the obese	Chapman, DG 2014	56	AMERICAN JOURNAL OF RESPIRATORY AND CRITICAL CARE MEDICINE
90	The estimation of lung mechanics parameters in the presence of pathology: A theoretical analysis	Bates, JHT 2006	56	RESPIROLOGY
91	Total respiratory impedance measured by means of the forced oscillation technique in subjects with and without respiratory complaints	Pasker, HG 1996	56	ANNALS OF BIOMEDICAL ENGINEERING
92	Comparison of oscillometry devices using active mechanical test loads	Dandurand, RJ 2019	55	EUROPEAN RESPIRATORY JOURNAL
93	Peripheral airway dysfunction and relationship with symptoms in smokers with preserved spirometry	Jetmalani, K 2018	55	ERJ OPEN RESEARCH

Rank	Title	First Author (Year)	Times Cited, WoS Core	Source Title
94	Quantification of heterogeneity in lung disease with image-based pulmonary function testing	Stahr, CS 2016	55	RESPIROLOGY
95	A comparison of lung function methods for assessing dose-response effects of salbutamol	Houghton, CM 2004	55	SCIENTIFIC REPORTS
96	Current place of impulse oscillometry in the assessment of pulmonary diseases	Bednarek, M 2020	54	BRITISH JOURNAL OF CLINICAL PHARMACOLOGY
97	Lung Function in South African Adolescents Infected Perinatally with HIV and Treated Long-Term with Antiretroviral Therapy	Pasker, HG 2017	54	RESPIRATORY MEDICINE
98	Comparison of bronchodilator response in patients with asthma and healthy subjects using spirometry and oscillometry	Dandurand, RJ 2011	54	ANNALS OF THE AMERICAN THORACIC SOCIETY
99	Changes in respiratory mechanics during cardiac surgery	Babik, B 2003	54	ANNALS OF ALLERGY ASTHMA & IMMUNOLOGY
100	MEASUREMENT OF RESPIRATORY SYSTEM RESISTANCE BY FORCED OSCILLATION IN NORMAL-CHILDREN - A COMPARISON WITH SPIROMETRIC VALUES	LEBECQUE, P 1991	54	ANESTHESIA AND ANALGESIA
